# Outcomes for hospitalized patients with idiopathic pulmonary fibrosis treated with antifibrotic medications

**DOI:** 10.1186/s12890-021-01607-2

**Published:** 2021-07-17

**Authors:** Bryan T. Kelly, Viengneesee Thao, Timothy M. Dempsey, Lindsey R. Sangaralingham, Stephanie R. Payne, Taylor T. Teague, Teng Moua, Nilay D. Shah, Andrew H. Limper

**Affiliations:** 1 Department of Pulmonary and Critical Care Medicine, Mayo Clinic, Gonda 18-South, 200 1st St SW, Rochester, MN 55905 USA; 2 Mayo Clinic Robert D. and Patricia E. Kern Center for the Science of Health Care Delivery, Rochester, MN USA; 3 OptumLabs, Cambridge, MA USA; 4 Division of Health Care Delivery Research, Mayo Clinic, Rochester, MN USA

**Keywords:** Idiopathic pulmonary fibrosis, Antifibrotics, Hospitalization, Critical care, Mechanical ventilation

## Abstract

**Background:**

Idiopathic Pulmonary Fibrosis is a chronic, progressive interstitial lung disease for which there is no cure. However, lung function decline, hospitalizations, and mortality may be reduced with the use of the antifibrotic medications, nintedanib and pirfenidone. Historical outcomes for hospitalized patients with Idiopathic Pulmonary Fibrosis are grim; however there is a paucity of data since the approval of nintedanib and pirfenidone for treatment. In this study, we aimed to determine the effect of nintedanib and pirfenidone on mortality following respiratory-related hospitalizations, intensive care unit (ICU) admission, and mechanical ventilation.

**Methods:**

Using a large U.S. insurance database, we created a one-to-one propensity score matched cohort of patients with idiopathic pulmonary fibrosis treated and untreated with an antifibrotic who underwent respiratory-related hospitalization between January 1, 2015 and December 31, 2018. Mortality was evaluated at 30 days and end of follow-up (up to 2 years). Subgroup analyses were performed for all patients receiving treatment in an ICU and those receiving invasive and non-invasive mechanical ventilation during the index hospitalization.

**Results:**

Antifibrotics were not observed to effect utilization of mechanical ventilation or ICU treatment during the index admission or effect mortality at 30-days. If patients survived hospitalization, mortality was reduced in the treated cohort compared to the untreated cohort when followed up to two years (20.1% vs 47.8%).

**Conclusions:**

Treatment with antifibrotic medications does not appear to directly improve 30-day mortality during or after respiratory-related hospitalizations. Post-hospital discharge, however, ongoing antifibrotic treatment was associated with improved long-term survival.

## Background

Idiopathic Pulmonary Fibrosis (IPF) is a chronic, progressive, fibrosing interstitial pneumonia characterized by progressive dyspnea and deteriorating lung function [1]. IPF occurs predominantly in older adults, particularly in men and patients with a history of cigarette smoking, and is defined by histopathologic and/or radiologic pattern of Usual Interstitial Pneumonia (UIP) [1]. The prognosis of IPF is poor overall with reported median survival between 2 and 5 years, though a significant proportion will survive for more than a decade [2, 3].

Over the years, various therapies have been studied for the treatment of IPF, yet none were found to offer benefit and recommendations were made against their use [4]. In October of 2014, the Federal Drug Administration (FDA) approved the use of nintedanib and pirfenidone for treatment of IPF in the United States (U.S.). At the time of approval, both drugs demonstrated a decrease in the rate of decline of Forced Vital Capacity (FVC) but no mortality benefit, while nintedanib also demonstrated an increase in time to first acute exacerbation (AExIPF) in one of its trial treatment arms [5, 6]. With these findings, the American Thoracic Society (ATS) clinical practice guidelines were updated, and a conditional recommendation was made for use of the antifibrotic drugs in the treatment of IPF [7]. Given lack of initial data to support a mortality benefit, debate was had as to whether these medications provided enough value for their use given the high costs of treatment and side effects; however, later data obtained from pooled analyses of the antifibrotic drug trials did suggest an additional mortality benefit [8–11]. This data was complemented by that of an Australian IPF Registry [12], and more recently, administrative data from a large United States cohort of commercially insured and Medicare Advantage enrollees which also demonstrated improved mortality on antifibrotic therapy [13].

Despite growing evidence that antifibrotic therapy may improve overall mortality in IPF patients, questions remain as to their impact on specific at-risk subpopulations. One such group of significant interest is patients who are hospitalized, and within that group, those that receive treatment in an Intensive Care Unit (ICU) with and without mechanical ventilation (MV) which may be delivered through both invasive and non-invasive methods. Early data regarding outcomes in these patients demonstrated high mortality [14–18], and the ATS clinical practice guidelines made a weak recommendation against the use of invasive MV in such patients [4]. Since that time, additional data has been published regarding hospitalization and critical illness in IPF patients suggesting lower, albeit still significant mortality [19–23]. A focus on acutely ill and hospitalized patients, however, has not been systematically studied since the availability of antifibrotic therapy. In this study, we utilized a large U.S. administrative claims-based database to evaluate the outcomes of treated and untreated IPF patients hospitalized for acute respiratory-related causes, including those who were cared for in an ICU with or without invasive or non-invasive MV. We hypothesized that antifibrotic therapy prior to hospitalization with an acute respiratory illness may offer a survival advantage compared to untreated patients.

## Methods

### Data source

We used deidentified administrative claims data from the OptumLabs Data Warehouse (OLDW). The OLDW contains claims-based information on individuals from all 50 states comprising all ages, ethnicities, and racial groups who are commercially insured or have Medicare Advantage [24]. Since the data are deidentified, this research is exempt from being considered human subjects research by both the Mayo Clinic Institutional Review Board and the NIH. Since the subjects are completely de-identified, it is impossible to re-contact these individuals and hence, the need for additional informed consent is also waived by the Mayo Clinic Institutional Review Board.

### Study populations

We included all adult patients who had their first respiratory hospitalization between January 1, 2015 and December 31, 2018. Respiratory hospitalizations were identified using the following *International Classification of Diseases, Ninth Edition* (ICD-9) diagnosis codes: 460–466, 470–478, 480–488, 490–496, 500–508, 510–519; and *International Classification of Diseases, 10th Edition* (ICD-10) diagnosis codes: J00-J06, J09-J18, J20-J22, J30-J47, J60-J70, J80-J86, J90-J94, J95.1-J95.8, and J96-J99. A similar approach to studying respiratory hospitalizations in IPF patients with ICD-9 codes has previously been performed [20, 25].

**Fig. 1:**
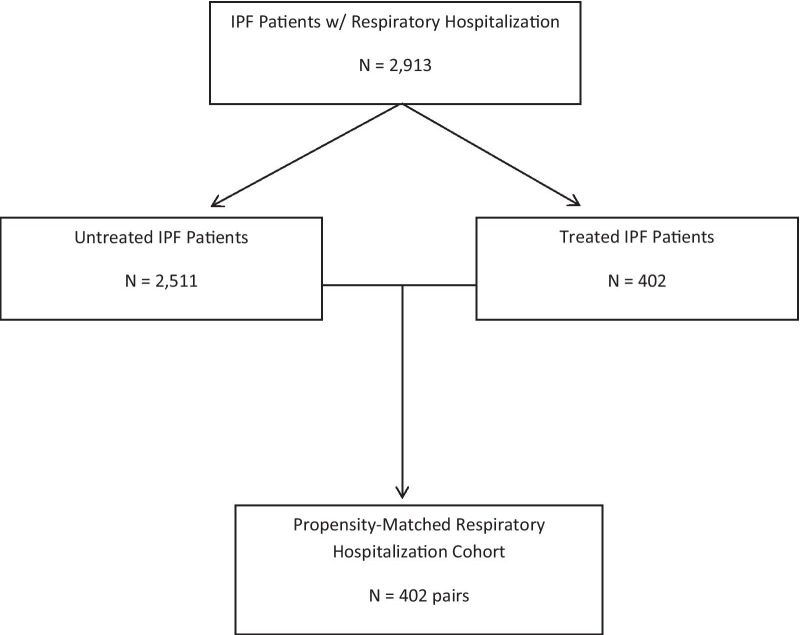
Generation of propensity-matched cohort of IPF patients with initial respiratory hospitalization

Patients were required to have a diagnosis of IPF (ICD-9: 516.31 or ICD-10: J84.112) prior to their index hospitalization and least 6 months of continuous enrollment in their health insurance plan before their hospitalization period. Patients without a diagnosis of IPF were dropped from our analysis. To further increase the accuracy of IPF identification, individuals with rheumatoid arthritis (240.9, 243, 244, 246.1, 246.8, E00-E03, E89.0), sarcoidosis (517.8, I35), and hypersensitivity pneumonitis (495.5, J67.9) were dropped from our analysis, along with individuals younger than 45 years (N = 48). We constructed treated and untreated cohorts of patients with IPF, and defined treated as any patient who filled a prescription for either pirfenidone or nintedanib at least 45 days prior to their index hospitalization. Those who did not fill a prescription for either pirfenidone or nintedanib at least 45 days prior to their index hospitalization were considered not treated. The process of cohort creation is presented in Fig. 1.

### Time on treatment

Patients were considered treated until they stopped filling a prescription for pirfenidone or nintedanib, or if there was a gap of 45 days or greater between their last treatment date and next fill date. We defined last treatment date as 30 days after the last fill date.

### Subgroup population

**Fig. 2:**
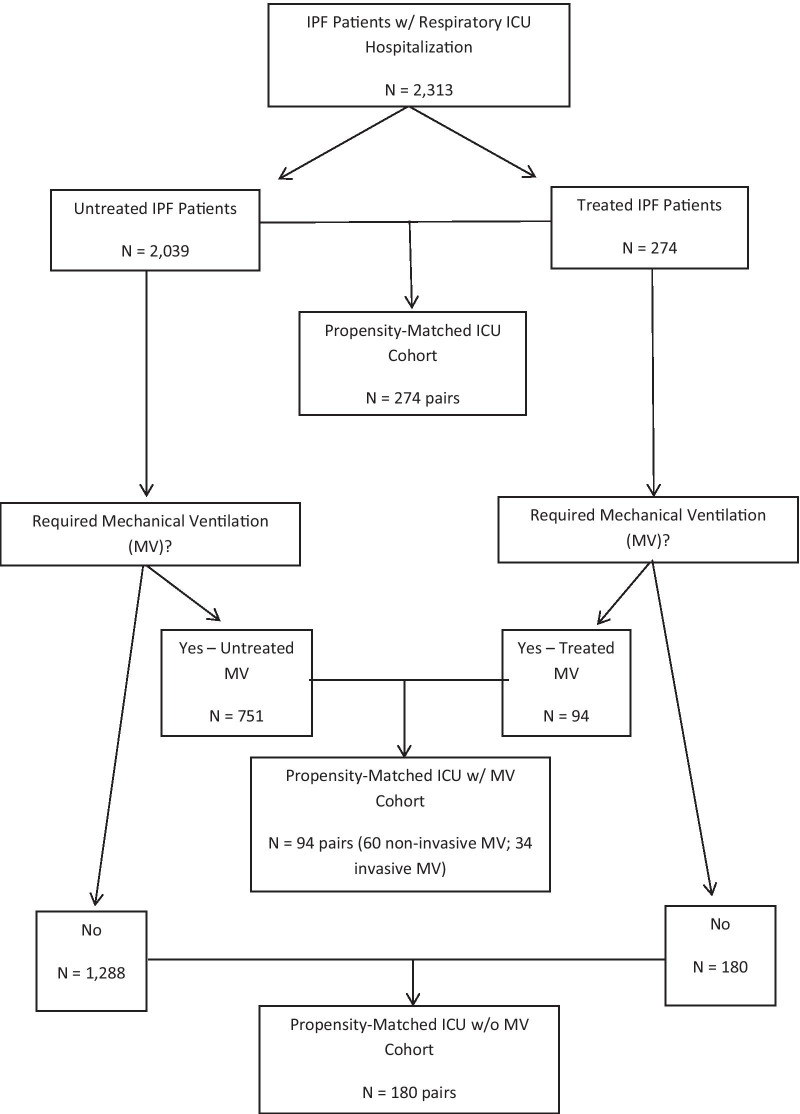
Generation of propensity-matched subgroups following initial intensive care unit hospitalization

We repeated the steps outlined previously to create three subgroups, indexed on a first respiratory-related hospitalization to an ICU. These included (1) all ICU respiratory hospitalizations; (2) those who had a respiratory-related ICU hospitalization requiring MV; and (3) those who had a respiratory-related ICU hospitalization without MV (Fig. 2). ICU hospitalizations were identified using revenue codes: 020X-021X. ICU hospitalizations with MV were identified using the revenue codes previously described and *Current Procedural Terminology* (CPT) codes: 94002-94005; or ICD-9 procedure codes: 96.70-96.72, 93.90; or ICD-10 procedure codes: 5A09357; 5A09457; 5A09557; 5A1935Z; 5A1945Z; 5A1955Z. We also conducted sensitivity analysis by comparing mortality outcomes among non-invasive MV (ICD-9 93.90 ICD-10 5A09357; 5A09457; 5A09557) and invasive MV (ICD-9 96.70-96.72and ICD-10 5A1935Z; 5A1945Z; 5A1955Z).

### Independent variables

We included the following patient demographics: age, sex, race/ethnicity, and census region. In addition, we included the year of index hospitalization and the reason for admission (i.e., primary diagnosis code on the medical claim). Smoking status (ICD-9: 649.0X, 305.1, 989.84, V15.82 and ICD10: F17.X, O00.33X, T65.2X, Z53.01, Z71.6, Z72.0, Z87.891), corticosteroid and oxygen use (CPT: E0424, E0425, E0430, E0431, E0433-E0435, E0440-E0447, E0455, E1352-E1354, E1356-E1359, E1391, E1392), and pulmonologist office visit (CPT: 99201-99205; 99211-99215; 99241-99245 with a specialty of ‘Pulmonary Disease’ listed), prior to the index hospitalization were also captured using their respective billing codes. Hospitalizations prior to the index hospitalization were also accounted for. Comorbidities were assessed using the Elixhauser comorbidity index and were captured with ICD-9 and ICD-10 diagnoses codes prior to the index hospitalization [26]. Prior data has shown that the Elixhauser comorbidity index correlates better with mortality than the Charlson comorbidity index in hospitalized patients with interstitial lung disease [27].

### Follow-up

Follow-up, in the treated cohort, started from the index hospitalization date and continued until the last treatment date, last date of enrollment in the health plan, death date, or end of the study period (April 1, 2019). Follow-up, in the untreated cohort, started from the index hospitalization date and continued until the last date of enrollment in health plan, death date, end of the study period, or start of pirfenidone or nintedanib.

### Study outcomes

The primary study outcome was all-cause mortality at 30 days from admission and at the end of follow-up. There are four main sources of mortality information in the OLDW: (1) the Social Security Administration Death Master; (2) electronic health records with deceased status written in the patient charts or patient family reports of death; (3) death as a reason for disenrollment in the health insurance plan; and (4) death indicated in the inpatient discharge status [28]. In a secondary analysis, we compared the use of ICU and MV for all hospitalized patients with IPF treated with or without antifibrotic therapy.

### Statistical analysis

We used propensity score matching to balance the differences in baseline characteristics between the treated and untreated cohorts. A propensity score was estimated using logistic regression based on age, sex, race, geographic region, year of index, reason for admission, smoking status, steroid and oxygen use, healthcare use (prior hospitalizations and pulmonary office visits), and comorbidities. Specifically, we used one-to-one nearest-neighbor caliper matching to match patients based on the logit of the propensity score [29]. We evaluated the standardized difference to assess the balance of covariates after matching, and a standardized difference ≤ 10% was considered acceptable [30]. When balance was not achieved through propensity score matching, we controlled for the unbalanced variable in the analysis.

We used logistic regression to compare mortality at 30 days from admission. Cox proportional hazards regression was used to compare time to death between treated and untreated patients following hospitalization [31]. A similar approach was used for each of the cohorts in the subgroup analysis, with logistic regression to compare ICU and MV use between treated patients and untreated patients during the index hospitalization. All analyses were conducted using SAS 9.4 (SAS Institute Inc.) and Stata version 15.1 (StataCorp).

## Results

### Baseline characteristics

We identified 402 treated and 2,511 untreated patients with IPF, who had a respiratory-related hospitalization between January 1, 2015 and December 31, 2018.The propensity-matched cohort included 402 matched pairs of treated and untreated patients (Table 1, Fig. 1). We then identified 274 treated and 2,039 untreated patients with IPF, who had an ICU hospitalization between January 1, 2015 and December 31, 2018 and created a propensity-matched cohort for all ICU hospitalizations consisting of 274 matched pairs of treated and untreated patients (Table 2, Fig. 2). Of those requiring an ICU hospitalization with MV, we identified 94 treated and 751 untreated patients with IPF and created a propensity-matched cohort consisting of 94 matched pairs (Table 3, Fig. 2). Of the 94 matched pairs, 34 required invasive MV and 60 required non-invasive MV. Of those requiring an ICU hospitalization without MV, we identified 180 treated and 1288 untreated patients with IPF and created a propensity-matched cohort of 180 matched (Table 4, Fig. 2).

**Table 1:** Baseline demographics of patients with idiopathic pulmonary fibrosis before and after propensity score matching – initial respiratory hospitalizations

	Before propensity score matching			After propensity score matching		
	No Rx (N = 2511)	Pirfenidone/Nintedanib (N = 402)	Std. Diff	No Rx (N = 402)	Pirfenidone/Nintedanib (N = 402)	Std. Diff
*Age group*						
45–64	339 (13.5%)	53 (13.2%)	− 0.009	55 (13.7%)	53 (13.2%)	− 0.015
65–74	713 (28.4%)	163 (40.5%)	0.258	162 (40.3%)	163 (40.5%)	0.005
75 +	1459 (58.1%)	186 (46.3%)	− 0.239	185 (46.0%)	186 (46.3%)	0.005
*Gender*						
Female	1182 (47.1%)	125 (31.1%)	− 0.332	132 (32.8%)	125 (31.1%)	− 0.037
Male	1329 (52.9%)	277 (68.9%)	0.332	270 (67.2%)	277 (68.9%)	0.037
*Race*						
White	1676 (66.7%)	293 (72.9%)	0.134	290 (72.1%)	293 (72.9%)	0.017
Black	365 (14.5%)	33 (8.2%)	− 0.200	40 (10.0%)	33 (8.2%)	− 0.061
Hispanic	311 (12.4%)	47 (11.7%)	− 0.021	48 (11.9%)	47 (11.7%)	− 0.008
Other	159 (6.3%)	29 (7.2%)	0.035	24 (6.0%)	29 (7.2%)	0.050
*Census region*						
Midwest	704 (28.0%)	110 (27.4%)	− 0.015	103 (26.5%)	110 (27.4%)	0.039
Northeast	399 (15.9%)	56 (13.9%)	− 0.055	61 (15.2%)	56 (13.9%)	− 0.035
South	1184 (47.2%)	201 (50.0%)	0.057	208 (51.7%)	201 (50.0%)	− 0.035
West	224 (8.9%)	35 (8.7%)	− 0.008	30 (7.5%)	35 (8.7%)	0.046
*Baseline comorbidities*						
Cardiac Arrhythmia	1037 (41.3%)	129 (32.1%)	− 0.192	113 (28.1%)	129 (32.1%)	0.087
Congestive Heart Failure	976 (38.9%)	108 (26.9%)	− 0.257	97 (24.1%)	108 (26.9%)	0.063
Other Chronic Pulmonary Conditions	1833 (73.0%)	260 (64.7%)	− 0.180	261 (65.0%)	260 (64.7%)	− 0.005
Depression	501 (20.0%)	72 (17.9%)	− 0.052	68 (16.9%)	72 (17.9%)	0.026
Diabetes	973 (38.7%)	146 (36.3%)	− 0.050	136 (33.8%)	146 (36.3%)	0.052
Hypertension	1934 (77.0%)	279 (69.4%)	− 0.173	275 (68.4%)	279 (69.4%)	0.021
Pulmonary Circulation Disorder	644 (25.6%)	112 (27.9%)	0.050	112 (27.9%)	112 (27.9%)	0.000
Renal Failure	603 (24.0%)	72 (17.9%)	− 0.150	70 (17.4%)	72 (17.9%)	0.013
Solid Tumor without Metastasis	347 (13.8%)	59 (14.7%)	0.025	65 (16.2%)	59 (14.7%)	− 0.041
Valvular Disease	642 (25.6%)	70 (17.4%)	− 0.199	74 (18.4%)	70 (17.4%)	− 0.026
*Elixhauser comorbidity index*						
Mean (SD)	5.7 (3.4)	4.7 (2.8)	− 0.344	4.5 (2.9)	4.7 (2.8)	0.062
Median	5.0	4.0	−	4.0	4.0	–
Q1, Q3	3.0, 8.0	3.0, 6.0	−	2.0, 6.0	3.0, 6.0	–
*N hospitalizations in baseline*						
0	1452 (57.8%)	315 (78.4%)	0.452	316 (78.6%)	315 (78.4%)	− 0.006
1	662 (26.4%)	70 (17.4%)	− 0.218	73 (18.2%)	70 (17.4%)	− 0.020
2 +	397 (15.8%)	17 (4.2%)	− 0.393	13 (3.2%)	17 (4.2%)	0.053
*Year of hospitalization*						
2015	561 (22.3%)	36 (9.0%)	− 0.375	37 (9.2%)	36 (9.0%)	− 0.009
2016	604 (24.1%)	115 (28.6%)	0.104	127 (31.6%)	115 (28.6%)	− 0.065
2017	661 (26.3%)	131 (32.6%)	0.138	118 (29.4%)	131 (32.6%)	0.070
2018	685 (27.3%)	120 (29.9%)	0.057	120 (29.9%)	120 (29.9%)	0.000
*Reason for admission*						
Diseases of respiratory system	1393 (55.5%)	158 (39.3%)	− 0.328	155 (38.6%)	158 (39.3%)	0.015
Diseases affecting the interstitium	928 (37.0%)	239 (59.5%)	0.462	243 (60.4%)	239 (59.5%)	− 0.020
All other reasons	190 (7.6%)	5 (1.2%)	− 0.312	4 (1.0%)	5 (1.2%)	0.024
Pulmonologist visit	1323 (52.7%)	323 (80.3%)	0.613	322 (80.1%)	323 (80.3%)	− 0.006
Smoker	1274 (50.7%)	198 (49.3%)	− 0.030	205 (51.1%)	198 (49.3%)	− 0.035
Steroid use	1351 (53.8%)	229 (57.0%)	0.064	227 (56.5%)	229 (57.0%)	0.010
Oxygen use	1482 (59.0%)	320 (79.6%)	0.424	310 (77.1%)	320 (79.6%)	0.006

**Table 2:** Subgroup analysis: baseline demographics of patients with idiopathic pulmonary fibrosis before and after propensity score matching – all intensive care unit respiratory hospitalizations

	Before propensity score matching			After propensity score matching		
	No Rx (N = 2039)	Pirfenidone/Nintedanib (N = 274)	Std. Diff	No Rx (N = 274)	Pirfenidone/Nintedanib (N = 274)	Std. Diff
*Age group*						
45–64	287 (14.1%)	39 (14.2%)	0.006	35 (12.8%)	39 (14.2%)	0.043
65–74	589 (28.9%)	111 (40.5%)	0.245	116 (42.3%)	111 (40.5%)	− 0.037
75 +	1163 (57.0%)	124 (45.3%)	− 0.238	123 (44.9%)	124 (45.3%)	0.007
*Gender*						
Female	905 (44.4%)	78 (28.5%)	− 0.336	85 (31.0%)	78 (28.5%)	− 0.056
Male	1132 (55.6%)	196 (71.5%)	0.336	189 (69.0%)	196 (71.5%)	0.056
*Race*						
White	1304 (64.0%)	179 (65.3%)	0.027	175 (63.9%)	179 (65.3%)	0.031
Black	168 (8.2%)	11 (4.0%)	− 0.177	13 (4.7%)	11 (4.0%)	− 0.036
Hispanic	218 (10.7%)	31 (11.3%)	0.020	27 (9.9%)	31 (11.3%)	0.047
Other	349 (17.1%)	53 (19.3%)	0.060	59 (21.5%)	53 (19.3%)	− 0.054
*Census region*						
Midwest	564 (27.7%)	71 (25.9%)	− 0.040	65 (23.7%)	71 (25.9%)	0.051
Northeast	286 (14.0%)	31 (11.3%)	− 0.082	38 (13.9%)	31 (11.3%)	− 0.077
South	1008 (49.4%)	143 (52.2%)	0.056	142 (51.8%)	143 (52.2%)	0.007
West	181 (8.9%)	29 (10.6%)	0.057	29 (10.6%)	29 (10.6%)	0.000
*Baseline comorbidities*						
Cardiac Arrhythmia	892 (43.7%)	108 (39.4%)	− 0.087	112 (40.9%)	108 (39.4%)	− 0.030
Congestive Heart Failure	845 (41.4%)	87 (31.8%)	− 0.201	97 (35.4%)	87 (31.8%)	− 0.077
Other Chronic Pulmonary Conditions	1568 (76.9%)	180 (65.7%)	− 0.250	185 (67.5%)	180 (65.7%)	− 0.039
Depression	407 (20.0%)	51 (18.6%)	− 0.035	42 (15.3%)	51 (18.6%)	0.087
Diabetes	819 (40.2%)	104 (38.0%)	− 0.044	109 (39.8%)	104 (38.0%)	− 0.037
Hypertension	1600 (78.5%)	194 (70.8%)	− 0.176	200 (73.0%)	194 (70.8%)	− 0.049
Pulmonary Circulation Disorder	553 (27.1%)	86 (31.4%)	0.093	81 (29.6%)	86 (31.4%)	0.040
Renal Failure	486 (23.8%)	52 (19.0%)	− 0.118	47 (17.2%)	52 (19.0%)	0.047
Solid Tumor without Metastasis	302 (14.8%)	40 (14.6%)	− 0.006	38 (13.9%)	40 (14.6%)	0.021
Valvular Disease	551 (27.0%)	58 (21.2%)	− 0.135	59 (21.5%)	58 (21.2%)	− 0.009
*Elixhauser comorbidity index*						
Mean (SD)	5.9 (3.3)	5.2 (2.9)	− 0.258	5.1 (3.2)	5.2 (2.9)	0.004
Median	6.0	5.0	−	5.0	5.0	−
Q1, Q3	3.0, 8.0	3.0, 7.0	−	3.0, 7.0	3.0, 7.0	−
*N hospitalizations in baseline*						
0	1132 (55.5%)	181 (66.1%)	0.217	178 (65.0%)	181 (66.1%)	0.023
1	535 (26.2%)	67 (24.5%)	− 0.042	70 (25.5%)	67 (24.5%)	− 0.025
2 +	372 (18.2%)	26 (9.5%)	− 0.255	26 (9.5%)	26 (9.5%)	0.000
*Year of hospitalization*						
2015	448 (22.0%)	30 (10.9%)	− 0.301	30 (10.9%)	30 (10.9%)	0.000
2016	498 (24.4%)	69 (25.2%)	0.019	72 (26.3%)	69 (25.2%)	− 0.025
2017	532 (26.1%)	84 (30.7%)	0.101	83 (30.3%)	84 (30.7%)	0.008
2018	561 (27.5%)	91 (33.2%)	0.124	89 (32.5%)	91 (33.2%)	0.016
*Reason for admission*						
Diseases of respiratory system	1352 (66.3%)	136 (49.6%)	− 0.342	136 (49.6%)	136 (49.6%)	0.000
Diseases affecting the interstitium	523 (25.6%)	132 (48.2%)	0.479	134 (48.9%)	132 (48.2%)	− 0.015
All other reasons	164 (8.0%)	6 (2.2%)	− 0.268	4 (1.5%)	6 (2.2%)	0.055
Pulmonologist visit	1136 (55.7%)	225 (82.1%)	0.595	230 (83.9%)	225 (82.1%)	− 0.049
Smoker	1066 (52.3%)	135 (49.3%)	− 0.060	134 (48.9%)	135 (49.3%)	0.007
Steroid use	1145 (56.2%)	183 (66.8%)	0.219	188 (68.6%)	183 (66.8%)	− 0.039
Oxygen use	1249 (61.3%)	228 (83.2%)	0.430	219 (79.9%)	228 (83.2%)	− 0.027

**Table 3:** Subgroup analysis: baseline demographics of patients with idiopathic pulmonary fibrosis before and after propensity score matching – intensive care unit respiratory hospitalizations requiring mechanical ventilation

	Before propensity score matching			After propensity score matching		
	No Rx (N = 751)	Pirfenidone/Nintedanib (N = 94)	Std. Diff	No Rx (N = 94)	Pirfenidone/Nintedanib (N = 94)	Std. Diff
*Age group*						
45–64	119 (15.8%)	15 (16.0%)	0.003	19 (20.2%)	15 (16.0%)	− 0.111
65–74	248 (33.0%)	46 (48.9%)	0.328	5 (47.9%)	46 (48.9%)	0.021
75 +	384 (51.1%)	33 (35.1%)	− 0.328	30 (31.9%)	33 (35.1%)	− 0.068
*Gender*						
Female	318 (42.3%)	26 (27.7%)	− 0.312	27 (28.7%)	26 (27.7%)	− 0.024
Male	433 (57.7%)	68 (72.3%)	0.312	67 (71.3%)	68 (72.3%)	0.024
*Race*						
White	470 (62.6%)	58 (61.7%)	− 0.018	61 (64.9%)	58 (61.7%)	− 0.066
Black	75 (10.0%)	6 (6.4%)	− 0.132	3 (3.2%)	6 (6.4%)	0.150
Hispanic	87 (11.6%)	15 (16.0%)	0.127	13 (13.8%)	15 (16.0%)	0.060
Other	119 (15.8%)	15 (16.0%)	0.003	17 (18.1%)	15 (16.0%)	− 0.057
*Census region*						
Midwest	178 (23.7%)	27 (28.7%)	0.114	30 (31.9%)	27 (28.7%)	− 0.069
Northeast	113 (15.0%)	9 (9.6%)	− 0.167	12 (12.8%)	9 (9.6%)	− 0.101
South	402 (53.5%)	50 (53.2%)	− 0.007	43 (45.7%)	50 (53.2%)	0.149
West	58 (7.7%)	8 (8.5%)	0.029	9 (9.6%)	8 (8.5%)	− 0.037
*Baseline comorbidities*						
Cardiac Arrhythmia	350 (46.6%)	30 (31.9%)	− 0.303	30 (31.9%)	30 (31.9%)	0.000
Congestive Heart Failure	337 (44.9%)	28 (29.8%)	− 0.315	25 (26.6%)	28 (29.8%)	0.071
Other Chronic Pulmonary Conditions	604 (80.4%)	65 (69.1%)	− 0.261	70 (74.5%)	65 (69.1%)	− 0.118
Depression	165 (22.0%)	22 (23.4%)	0.034	23 (24.5%)	22 (23.4%)	− 0.025
Diabetes	310 (41.3%)	36 (38.3%)	− 0.061	38 (40.4%)	36 (38.3%)	− 0.043
Hypertension	604 (80.4%)	65 (69.1%)	− 0.261	64 (68.1%)	65 (69.1%)	0.023
Pulmonary Circulation Disorder	216 (28.8%)	27 (28.7%)	− 0.001	25 (26.6%)	27 (28.7%)	0.047
Renal Failure	184 (24.5%)	13 (13.8%)	− 0.273	14 (14.9%)	13 (13.8%)	− 0.030
Solid Tumor without Metastasis	119 (15.8%)	10 (10.6%)	− 0.154	9 (9.6%)	10 (10.6%)	0.035
Valvular Disease	207 (27.6%)	19 (20.2%)	− 0.173	20 (21.3%)	19 (20.2%)	− 0.026
*Elixhauser comorbidity index*						
Mean (SD)	6.3 (3.3)	4.9 (3.0)	− 0.414	5.1 (3.2)	4.9 (3.0)	− 0.061
Median	6.0	5.0	−	4.5	5.0	−
Q1, Q3	4.0, 9.0	3.0, 6.0	−	3.0, 8.0	3.0, 6.0	−
*N hospitalizations in baseline*						
0	383 (51.0%)	61 (64.9%)	0.284	54 (57.4%)	61 (64.9%)	0.153
1	211 (28.1%)	24 (25.5%)	− 0.058	25 (26.6%)	24 (25.5%)	− 0.024
2 +	157 (20.9%)	9 (9.6%)	− 0.319	15 (16.0%)	9 (9.6%)	− 0.192
*Year of hospitalization*						
2015	176 (23.4%)	12 (12.8%)	− 0.280	10 (10.6%)	12 (12.8%)	0.066
2016	204 (27.2%)	30 (31.9%)	0.104	29 (30.9%)	30 (31.9%)	0.023
2017	200 (26.6%)	27 (28.7%)	0.047	31 (33.0%)	27 (28.7%)	− 0.092
2018	171 (22.8%)	25 (26.6%)	0.089	24 (25.5%)	25 (26.6%)	0.024
*Reason for admission*						
Diseases of respiratory system	562 (74.8%)	47 (50.0%)	− 0.530	40 (42.6%)	47 (50.0%)	0.150
Diseases affecting the interstitium	144 (19.2%)	44 (46.8%)	0.615	49 (52.1%)	44 (46.8%)	− 0.107
All other reasons	45 (6.0%)	3 (3.2%)	− 0.134	5 (5.3%)	3 (3.2%)	− 0.106
Pulmonologist visit	408 (54.3%)	80 (85.1%)	0.709	85 (90.4%)	80 (85.1%)	− 0.162
Smoker	414 (55.1%)	46 (48.9%)	− 0.124	52 (55.3%)	46 (48.9%)	− 0.127
Steroid use	420 (55.9%)	69 (73.4%)	0.371	69 (73.4%)	69 (73.4%)	0.000
Oxygen use	484 (64.4%)	83 (88.3%)	0.433	74 (78.7%)	83 (88.3%)	0.053

**Table 4:** Subgroup analysis: baseline demographics of patients with idiopathic pulmonary fibrosis before and after propensity score matching – intensive care unit respiratory hospitalizations without mechanical ventilation

	Before propensity score matching			After propensity score matching		
	No Rx (N = 1288)	Pirfenidone/Nintedanib (N = 180)	Std. Diff	No Rx (N = 180)	Pirfenidone/Nintedanib (N = 180)	Std. Diff
*Age group*						
45–64	168 (13.0%)	24 (13.3%)	0.010	30 (16.7%)	24 (13.3%)	− 0.093
65–74	341 (26.5%)	65 (36.1%)	0.208	65 (36.1%)	65 (36.1%)	0.000
75 +	779 (60.5%)	91 (50.6%)	− 0.201	85 (47.2%)	91 (50.6%)	0.067
*Gender*						
Female	587 (45.6%)	52 (28.9%)	− 0.352	55 (30.6%)	52 (28.9%)	− 0.036
Male	699 (54.4%)	128 (71.1%)	0.352	125 (69.4%)	128 (71.1%)	0.036
*Race*						
White	834 (64.8%)	121 (67.2%)	0.050	118 (65.6%)	121 (67.2%)	0.035
Black	93 (7.2%)	5 (2.8%)	− 0.205	7 (3.9%)	5 (2.8%)	− 0.062
Hispanic	131 (10.2%)	16 (8.9%)	− 0.044	17 (9.4%)	16 (8.9%)	− 0.019
Other	230 (17.9%)	38 (21.1%)	0.086	38 (21.1%)	38 (21.1%)	0.000
*Census region*						
Midwest	386 (30.0%)	44 (24.4%)	− 0.125	40 (22.2%)	44 (24.4%)	0.053
Northeast	173 (13.4%)	22 (12.2%)	− 0.037	27 (15.0%)	22 (12.2%)	− 0.081
South	606 (47.0%)	93 (51.7%)	0.094	90 (50.0%)	93 (51.7%)	0.033
West	123 (9.5%)	21 (11.7%)	0.068	23 (12.8%)	21 (11.7%)	− 0.034
*Baseline comorbidities*						
Cardiac Arrhythmia	542 (42.1%)	78 (43.3%)	0.027	80 (44.4%)	78 (43.3%)	− 0.022
Congestive Heart Failure	508 (39.4%)	59 (32.8%)	− 0.137	63 (35.0%)	59 (32.8%)	− 0.047
Other Chronic Pulmonary Conditions	964 (74.8%)	115 (63.9%)	− 0.240	118 (65.6%)	115 (63.9%)	− 0.035
Depression	242 (18.8%)	29 (16.1%)	− 0.071	27 (15.0%)	29 (16.1%)	0.031
Diabetes	509 (39.5%)	68 (37.8%)	− 0.034	69 (38.3%)	68 (37.8%)	− 0.011
Hypertension	996 (77.3%)	129 (71.7%)	− 0.129	132 (73.3%)	129 (71.7%)	− 0.037
Pulmonary Circulation Disorder	337 (26.2%)	59 (32.8%)	0.144	62 (34.4%)	59 (32.8%)	− 0.035
Renal Failure	302 (23.4%)	39 (21.7%)	− 0.042	37 (20.6%)	39 (21.7%)	0.027
Solid Tumor without Metastasis	183 (14.2%)	30 (16.7%)	0.067	27 (15.0%)	30 (16.7%)	0.046
Valvular Disease	344 (26.7%)	39 (21.7%)	− 0.115	40 (22.2%)	39 (21.7%)	− 0.013
*Elixhauser comorbidity index*						
Mean (SD)	5.8 (3.2)	5.3 (2.8)	− 0.166	5.4 (3.0)	5.3 (2.8)	− 0.050
Median	5.0	5.0	−	5.0	5.0	−
Q1, Q3	3.0, 8.0	3.0, 7.0	−	3.0, 7.0	3.0, 7.0	−
*N hospitalizations in baseline*						
0	749 (58.2%)	120 (66.7%)	0.176	115 (63.9%)	120 (66.7%)	0.058
1	324 (25.2%)	43 (23.9%)	− 0.030	43 (23.9%)	43 (23.9%)	0.000
2 +	215 (16.7%)	17 (9.4%)	− 0.215	22 (12.2%)	17 (9.4%)	− 0.089
*Year of hospitalization*						
2015	272 (21.1%)	18 (10.0%)	− 0.311	17 (9.4%)	18 (10.0%)	0.019
2016	294 (22.8%)	39 (21.7%)	− 0.025	36 (20.0%)	39 (21.7%)	0.041
2017	332 (25.8%)	57 (31.7%)	0.130	61 (33.9%)	57 (31.7%)	− 0.047
2018	390 (30.3%)	66 (36.7%)	0.135	66 (36.7%)	66 (36.7%)	0.000
*Reason for admission*						
Diseases of respiratory system	790 (61.3%)	89 (49.4%)	− 0.240	85 (47.2%)	89 (49.4%)	0.044
Diseases affecting the interstitium	379 (29.4%)	88 (48.9%)	0.406	91 (50.6%)	88 (48.9%)	− 0.033
All other reasons	119 (9.2%)	3 (1.7%)	− 0.399	4 (2.2%)	3 (1.7%)	− 0.040
Pulmonologist visit	728 (56.5%)	145 (80.6%)	0.535	150 (83.3%)	145 (80.6%)	− 0.072
Smoker	652 (50.6%)	89 (49.4%)	− 0.024	92 (51.1%)	89 (49.4%)	− 0.033
Steroid use	725 (56.3%)	114 (63.3%)	0.144	114 (63.3%)	114 (63.3%)	− 0.000
Oxygen use	765 (59.4%)	145 (80.6%)	0.431	143 (79.4%)	145 (80.6%)	− 0.041

After propensity matching, baseline characteristics were well balanced between the respiratory-related hospitalization cohorts as shown in Table 1. Furthermore, baseline characteristics were well balanced between the ICU MV subgroups as presented in Tables 2 and 4, respectively. Baseline characteristics within the MV subgroup were well balanced with the exception of: age, race, census region, baseline comorbidities, number of baseline hospitalizations, and smoking status as shown in Table 3.

### All-cause mortality

**Fig. 3:**
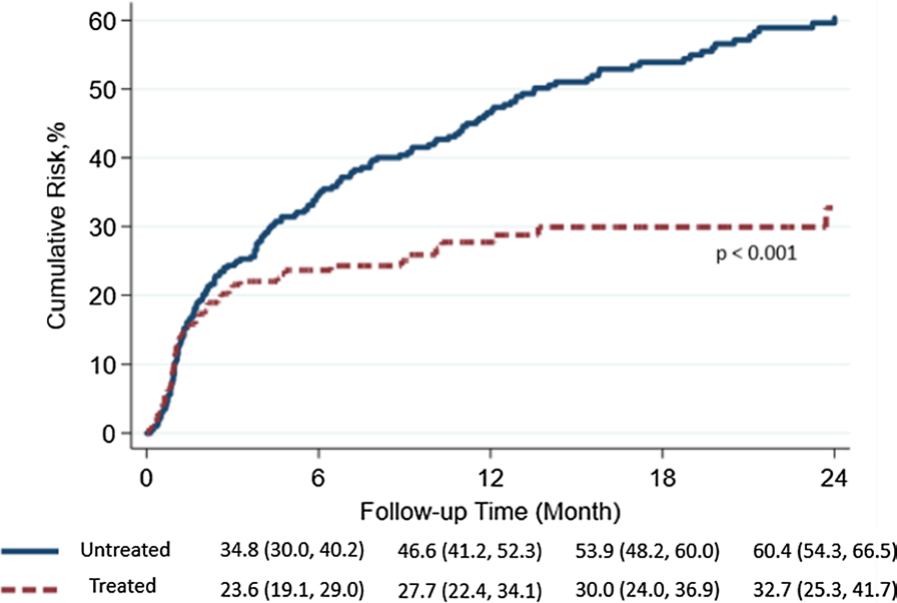
Mortality cumulative risk following initial respiratory hospitalization in patients on treatment for idiopathic pulmonary fibrosis compared with untreated matched cohort

There was no difference in 30-day mortality between the treated and untreated cohort; 10.0% vs. 10.2%, hazard ratio (HR) 0.96, 95% confidence interval (CI) 0.70–1.33, *p* value = 0.812, however antifibrotic treatment prior to any hospitalization was associated with a lower risk of all-cause mortality [treated cohort: 39 per 100 person-years vs untreated cohort: 55 per 100 person-years; HR: 0.59, 95% CI 0.45–0.77, *p* < 0.001] when followed through the first 2 years of follow-up as shown in Table 5 and Fig. 3.

**Table 5:** Mortality at 30 days and end of follow-up following respiratory hospitalizations, ICU hospitalizations, and ICU hospializations with and without mechanical ventilation

	Untreated	Treated	Statistical Analysis
*All respiratory hospitalizations*	N = 402	N = 402	
30 day mortality, total (%)	40 (10.0)	41 (10.2)	HR 0.96 (CI 0.70–1.33), *p* = 0.812
End of follow-up mortality, total (%)	192 (47.8)	81 (20.1)	HR 0.59 (CI 0.45–0.77), *p* < 0.001
Months of follow-up mean(SD)	10.6 (12.1) Median: 5.3	6.3 (8.9) Median: 2.2	
*ICU hospitalizations*	N = 274	N = 274	
30 day mortality, total (%)	45 (16.4)	49 (17.9)	HR 1.05 (CI 0.71–1.58) *p* = 0.782
End of follow-up mortality, total (%)	140 (51.1)	102 (37.2)	HR 0.79 (CI 0.61–1.02) *p* = 0.075
Months of follow-up mean (SD)	8.7 (11.5) Median: 3.1	7.2 (10.4) Median: 2.4	
*ICU Hospitalizations w/all mechanical ventilation*	N = 94	N = 94	
30 day mortality, total (%)	29 (30.9)	27 (28.7)	HR 0.91 (CI 0.52–1.59) *p* = 0.734
End of follow-up mortality, total (%)	66 (70.2)	50 (53.2)	HR 0.64 (CI 0.43–0.94) *p* = 0.021
Months of Follow-up mean (SD)	6.3 (10.7) Median: 1.18	4.1 (6.2) Median: 1.55	
*ICU hospitalizations w/non-invasive mechanical ventilation*	N = 60	N = 60	
30 day mortality, total (%)	13 (21.7)	13 (21.7)	HR 0.94 (CI 0.34–2.57) *p* = 0.907
End of follow-up mortality, tota l(%)	39 (65.0)	26 (43.3)	HR 0.80 (CI 0.43–1.47) *p* = 0.472
Months of Follow-up mean (SD)	7.13 (11.02) Median: 2.37	4.73 (6.71) Median: 1.87	
*ICU hospitalizations w/invasive mechanical ventilation*	N = 34	N = 34	
30 day mortality, total (%)	10 (29.4)	14 (41.2)	HR 1.78 (CI 0.55–5.79) *p* = 0.339
End of follow-up mortality, total (%)	26 (76.5)	24 (70.6)	HR 1.47 (CI 0.66–3.27) *p* = 0.345
Months of Follow-up mean (SD)	8.25 (13.95) Median: 1.18	3.03 (5.11) Median: 1.05	
*ICU hospitalizations w/out mechanical ventilation*	N = 180	N = 180	
30 day mortality, total (%)	21 (11.7)	22 (12.2)	HR 1.00 (CI 0.56–1.83) *p* = 0.980
End of follow-up mortality, total (%)	79 (43.9)	52 (28.9)	HR 0.71 (CI 0.50–1.00) *p* = 0.055
Months of Follow-up mean (SD)	10.0 (11.5) Median: 5.9	8.8 (11.7) Median: 3.0

### All-cause mortality in the subgroup analysis

We found no difference in all-cause mortality following ICU hospitalizations at 30 days [16.4% treated vs 17.9% untreated, HR 1.05; CI 0.71–1.58, *p *value = 0.782] or at 2 years [51.1% treated vs 37.2% untreated, HR 0.79; CI 0.61–1.02, *p *value = 0.075] among those treated vs. not (Table 6). There was also no difference in 30-day mortality following MV [30.9% treated vs 28.7% untreated, HR 0.91; CI 0.52–1.59, *p *value = 0.734] however antifibrotic treatment was associated with a lower risk of all-cause mortality [70.2% treated vs 53.2% untreated, HR 0.64; CI 0.43–0.94, *p *value = 0.021]. When we repeated the analysis by invasive and non-invasive MV, we found no difference in 30-day mortality and 2 year mortality. We found no difference in 30-day mortality following ICU hospitalizations not requiring MV [11.7% treated vs 12.2% untreated, HR 1.00; CI 0.56–1.83, *p *value = 0.980], or at 2 years [43.9% treated vs 28.9% untreated, HR 0.71; CI 0.50–1.00, *p *value = 0.055] through 2 years of follow-up.

**Table 6:** Utilization of intensive care unit and mechanical ventilation during initial hospitalization

	Unmatched		Matched		Treated adjusted odds ratio [95% Confidence Interval]	*p* value
	Untreated N = 2,511	Treated N = 402	Untreated N = 402	Treated N = 402		
ICU	1385 (55.2%)	218 (54.2%)	224 (55.7%)	218 (54.2%)	0.94 [0.71–1.24]	0.67
All mechanical ventilation	407 (16.2%)	66 (16.4%)	58 (14.4%)	66 (16.4%)	1.17 [0.79–1.70]	0.44
Non-invasive MV	224 (8.9%)	37 (9.2%)	34 (8.5%)	37 (9.2%)	1.10 [0.67–1.79]	0.71
Invasive MV	183 (7.3%)	29 (7.2%)	24 (6.0%)	29 (7.2%)	1.22 [0.70–2.14]	0.48

### ICU and MV use

We found no difference in ICU admissions during initial hospitalization [OR 0.94, 95% CI 0.71–1.24, *p *value = 0.67] between treated and untreated patients (Table 5); and no difference in MV use [OR 1.17, 95% CI 0.79–1.70, *p *value = 0.44] (Table 6).

## Discussion

To our knowledge, this is the first use of real-world data to evaluate the effects of antifibrotics on hospitalization outcomes in patients with IPF. Our findings are unique as previously published data regarding IPF respiratory hospitalizations have not accounted for the impact of antifibrotic therapy, with much of the available data arising from tertiary referral centers where the acuity of cases and the care administered may limit generalizability.

We observed no impact on 30-day mortality following respiratory-related hospitalizations for patients with IPF treated with antifibrotic medications, including those who required ICU care for any reason, with or without MV, compared to a propensity matched untreated cohorts. Similar rates of ICU utilization across the cohort suggested treatment with antifibrotics prior to hospitalization did not reduce the acuity of hospitalizations. However, if patients survived hospitalization, those with ongoing antifibrotic treatment had improved survival compared to their untreated counterparts up to two years (Fig. 3).

As previously observed by Dempsey et al., treatment with antifibrotic medications is associated with a reduction in hospitalizations [13]. Though lacking a direct impact on 30-day mortality following hospitalizations, it might be suggested that antifibrotic therapy indirectly reduced mortality by reducing the number of hospitalizations. Until an intervention that improves outcomes during hospitalization is identified, prevention of decompensations leading to hospitalization remains an important mechanism to improving overall mortality outcomes.

Previous outcomes data for hospitalized IPF patients have shown in-hospital mortality rates ranging from 10.3 to 22.4%, though such studies were done either entirely prior to the wide availability of antifibrotic therapy [19, 21–23] or overlapping the time period before and after approval without specifically focusing on these therapies (Alqalyoobi). Two of these studies were performed using nationwide databases; the first from Rush et al. reviewed data from 2006 to 2012 and found an in-hospital mortality rate of 11.3% while Durheim et al. found an in-hospital mortality rate of 10.3% between October 2011 and October 2014 [21, 22]. While reporting similar mortality numbers to these others and our studies, Alqalyoobi et al. reported mortality differences between academic and non-academic institutions which may be of further consideration and interest [23]. An additional study from Brown et al. was a retrospective review of patients hospitalized at a tertiary care center between 1997 and 2012, and found a mortality of 22.4% [19]. In review, in-hospital mortality rates between the previously published database studies and our 30-day mortality rates were similar, supporting our study observation that antifibrotics do not appear to reduce in-hospital mortality. While our identified mortality differs from that found by Brown et al., it is important to note that this data was taken from a tertiary care center where acuity of cases, care delivered, and diagnostic techniques may differ compared to that in our population which includes care centers of all levels. Another outcome study from a nationwide Japanese database between 2010 and 2013 demonstrated an in-hospital mortality of 23%, twice that of U.S. national database studies [32]. Ethnic differences in IPF are controversial, and ethnicity alone cannot account for this discrepancy; however such factors, as well as exclusion of less severe cases in some East Asian insurance datasets, may partially explain the higher Japanese mortality [33, 34]. Furthermore, while the use or efficacy of the antifibrotic medications was not reported in this study, it should be noted that these medications were available for use in Japan during this period, further complicating a direct comparison to this population.

Outcomes regarding those receiving ICU care appear more favorable than previously described. Early literature regarding outcomes of critically ill IPF patients involved smaller cohorts from individual tertiary care centers, demonstrating very high in-hospital and short term mortality, as high as 100% when MV was utilized [14–18]. Over time, while remaining high, mortality has decreased as reported by recent multiple large cohort database studies demonstrating mortality or surrogate markers such as lung transplant falling closer to 50% [20–22]. The lower mortality rates observed in our study may possibly reflect improvements in the care of ICU patients, including advances in MV such as focused prevention of ventilator induced lung injury via use of “lung-protective ventilation” which include such factors as low tidal volume strategies, mechanical power, driving pressure, and stress index, as examples of the more nuanced approaches that may influence the lower mortality seen in our study [35–37]. Another factor that may contribute to lower ICU mortality may be lower thresholds for ICU admission among individual institutions. Increased recognition of AExIPF and advances in the delivery of respiratory support with non-invasive mechanical ventilation, including high flow oxygen, may allow avoidance of invasive MV, but still necessitate ICU admission. It might be expected that this population of patients would have a lower mortality than those treated with mechanical ventilation, however our findings did not suggest this, though factors that are difficult or impossible to account for such as patient preference and advanced care planning leave this an open area of interest.

In the context of our previous work on antifibrotics and treatment-related mortality, antifibrotics appear to have a role in reducing overall mortality in the first two years of therapy as well as reduce the number of hospitalizations, but do not appear to reduce in-hospital or 30-day mortality. Based on the current study findings, it would be reasonable to extrapolate that the introduction of antifibrotics during hospitalization would not affect in-hospital or 30 day outcomes, though further prospective studies are needed to clarify this.

While our earlier work showed no difference in overall mortality outcomes between the two treatment options, our current work could not sufficiently evaluate this [13]. A recent analysis of Medicare beneficiaries with IPF treated with antifibrotics shortly after FDA approval found a protective effect of pirfenidone on hospitalization rates [38]. Our initial study showed lower rates of hospitalization when antifibrotic medications were used but did not directly compare this outcome between the two therapies [13]. We feel that analyzing differences between these medications to help guide clinicians in management and advances in therapy is of great interest and should be continued.

There are several limitations to our study. First, while the use of administrative billing codes has been previously used to evaluate epidemiologic outcomes of IPF, it has been noted that such methodology risks misidentification of IPF patients [39–42]. To address this limitation, we identified IPF patients using the most specific available billing codes (ICD-9 516.31 and ICD-10 J84.112) as previously described [13]. Such method importantly does not include the codes for postinflammatory fibrosis (ICD-9 515 and ICD-10 J84.10) which have previously been utilized other in studies, but not found to be specific for IPF [43]. Additionally, we attempted to remove potentially confounding or inconsistent diagnoses by removing patients with diagnostic codes for rheumatoid arthritis, hypersensitivity pneumonitis, and sarcoidosis. Another billing code limitation is that of respiratory failure which may occur in acute, chronic, or acute on chronic presentation, with coding likely to be variable across institutions and providers. An additional impact of using administrative billing codes for patient identification is that only prevalence diagnoses can be identified with accuracy. While there are likely many patients with initial, or incident, diagnoses included in our study, the potential of changing insurance coverage leaves us only able to identify the time they meet IPF diagnosis criteria based upon billing codes under their current coverage.

Another limitation is our patient population was derived from a cohort of Medicare Advantage or private insurances with pharmaceutical benefits. This could limit generalizability to patients with different or complete lack of healthcare coverage, also perhaps associated with socioeconomic risk factors that may contribute to different outcomes. Furthermore, the impact of health insurance coverage on outcomes such as mortality is difficult to evaluate, with some evidence that those on Medicare coverage may have reduced in-hospital mortality [44, 45].

A third potential limitation is the use of prescription fills as a surrogate for adherence to antifibrotic medications. To ameliorate this, we only selected patients for the treatment arm that had continuous refilling of their prescriptions pre and post hospitalization as a surrogate of medication adherence. An additional limitation in our study is the difficulty in accurately identifying in-hospital mortality as this is protected health information. Given this challenge, we instead focused on short-term mortality at 30 days noting that deaths at this point in time would have either died while hospitalized or shortly after discharge.

An interesting question yet to be answered regarding hospitalizations that our data was not able to address is the mortality of AExIPF as there is no specific billing code for this condition. It is possible that many of the respiratory events that led to hospitalization could be classified as an AExIPF, but given the lack of a diagnostic code, this was unable to be evaluated. This leaves unanswered questions about whether the antifibrotics have any impact on AExIPF mortality as well as mortality of non-AExIPF respiratory hospitalizations. Additionally, diagnostic criteria for AExIPF were revised in 2016 [46], a retrospective review of such events overlapping this time period would pose additional challenges.

## Conclusions

In summary, our analysis of real-world patients with IPF hospitalized for respiratory-related conditions observed that pre-hospitalization treatment with antifibrotics had no impact on 30-day hospital-related mortality. If hospitalization was not fatal, however, ongoing treatment afterwards was associated with improved survival up to two years. Antifibrotics have previously been observed to reduce overall all-cause mortality and hospitalizations in IPF, and appear to sustain such effects even after hospitalization. While large retrospective observational studies such as ours provide broad and real-world observations, further research, particularly in the form of prospective and well documented observational studies, are necessary to identify predictive variables and interventions with a direct effect on mortality.

## Abbreviations

AExIPF: Acute exacerbation of IPF
ATS: American Thoracic Society
CI: Confidence interval
CPT: Current procedural terminology
FDA: Federal drug administration
FVC: Forced vital capacity
HR: Hazard ratio
ICD-9: International classification of diseases, ninth edition
ICD-10: International classification of diseases, tenth edition
ICU: Intensive care unit
IPF: Idiopathic pulmonary fibrosis
MV: Mechanical ventilation
OLDW: OptumLabs data warehouse
UIP: Usual interstitial pneumonia
US: United States
